# Work Inside Ocean Freight Containers—Personal Exposure to Off-Gassing Chemicals

**DOI:** 10.1093/annhyg/met033

**Published:** 2013-07-03

**Authors:** Urban Svedberg, Gunnar Johanson

**Affiliations:** ^1^Occupational and Environmental Medicine, Sundsvall Hospital, SE85186 Sundsvall, Sweden;; ^2^Work Environment Toxicology, Karolinska Institutet, IMM, S-17177 Stockholm, Sweden

**Keywords:** confined space, exposure assessment, fumigation, prevention, sea container

## Abstract

More than 500 million ocean freight container units are shipped annually between countries and continents. Residual levels of fumigants, as well as naturally occurring off-gassing chemicals emanating from the goods, constitute safety risks, which may affect uniformed workers upon entering the container. The aim of this study was to assess workers’ exposure during stripping of containers and is the first study of its kind. First, an experimental tracer gas method was investigated to determine its usefulness to approximate real exposures from gaseous fumigants and off-gassing volatile organic compounds (VOCs). Nitrous oxide was injected and left to distribute in the closed containers. The distribution of the tracer gas and initial (arrival) concentrations of off-gassing volatiles were measured prior to opening the containers. Second, personal exposure (breathing zone) and work zone air monitoring of both tracer gas and VOCs were carried out during stripping. Adsorbent tubes, bag samples, and direct-readings instruments (photoionization detector and Fourier transform infrared spectrometry) were used. The distribution studies with nitrous oxide, and the high correlation between the former and VOCs (*r* ^2^ ~ 0.8) during stripping, showed that the tracer gas method may well be used to approximate real exposures in containers. The average breathing zone and work zone concentrations during stripping of naturally ventilated 40-foot containers were 1–7% of the arrival concentrations; however, peaks up to 70% were seen during opening. Even if average exposures during stripping are significantly lower than arrival concentrations, they may still represent serious violations of occupational exposure limits in high-risk containers. The results from this and previous studies illustrate the need to establish practices for the safe handling of ocean freight containers. Until comprehensive recommendations are in place, personnel that need to enter such containers should, in addition to appropriate personal protective equipment, have access to equipment for measuring contaminants and for applying forced ventilation when necessary.

## INTRODUCTION

Globalization of trade has increased the volume of goods transported by ocean freight containers. In 2010, the worldwide container port throughput was 540 million units, that is the total number of containers handled by ports annually, expressed in 20-foot equivalent units (UNCTAD, 2012).

Goods, packaging, and wood pallets shipped in ocean freight containers may require voluntary or mandatory fumigation with gaseous pesticides to prevent pests and microbiological attacks on the goods and to stop their spread between countries and continents. Goods and packaging material themselves may also emit harmful volatile chemicals, which either occur naturally or remain after the production process, all of which will accumulate in the air inside the closed container. Residual levels of fumigants and off-gassing chemicals constitute health risks, which may affect unprepared workers upon entering the container. Many harmful substances, e.g. carbon monoxide, benzene, and phosphine, do not carry with them distinct inherent warning characteristics such as unpleasant odour or irritative properties.

Methyl bromide, sulphuryl fluoride, phosphine, chloropicrin, and hydrogen cyanide are examples of typical fumigation chemicals. Methyl bromide is being phased out but until substitutes are available for all situations in which it is currently employed it may still be used in accordance with the International Standards for Phytosanitary Measure, ISPM 15 protocol (FAO, 2009).

Fumigation can either be performed by pre-shipping application and ventilation (methyl bromide, sulphuryl fluoride) or by using an in-transit dose that maintains an effective level during transport (phosphine). Phosphine treatment is mostly carried out by placing small packages containing a powder or pellets of metallic phosphide, typically aluminium phosphide inside the container. During transportation, the phosphide reacts with atmospheric water vapour to form toxic phosphine gas and leaves a trace of solid harmless aluminium hydroxide (Windholz, 1983).

Fumigated containers that have not been ventilated before they are loaded on board fall within the scope of the International Maritime Dangerous Goods Code published by the International Maritime Organization (IMO, 2012). Such containers must carry warning signs, according to chapter 5.5 of the Code, indicating the chemical used and date of treatment. They must also be accompanied by correct transportation documents specifying the fumigation procedures. Detailed information is published in the IMO Recommendations covering the safe use of pesticides in ships, applicable to the fumigation of cargo transport units (IMO, 2008). Interestingly, the very same recommendation states that there is widespread non-compliance regarding signage and required documentation. Such lack of compliance raises serious concerns, as the warning sign is the first, and perhaps only, message the worker receives that the container air could be hazardous.

Stripping, a term used for unloading the goods inside a container, is normally done in container terminals and warehouses strategically located in various parts of a country. The content is typically stripped, rearranged, and reloaded onto trucks for delivery to retail outlets. A large number of warehouse workers are engaged on a daily basis in stripping and may spend several hours each day inside containers. When preparing for this study, we identified serious shortcomings in work routines and our impression is that the most common practice is to require uninformed and unprotected workers to strip the containers without knowing anything about their contents with respect to fumigants and off-gassing chemicals.

Occupational groups such as coast guards, customs officers, and food inspectors may be exposed to high levels of air contaminants as they enter unventilated containers to inspect cargo and hollow spaces, at times even crawling in the narrow space between the goods and the container ceiling. Down the distribution line, there is a low-grade exposure risk among retail personnel, consumers, and others handling goods that have been shipped in affected containers. Experimental studies have shown that methyl bromide, chloropicrin, and sulphuryl fluoride may absorb in consumer goods followed by slow release over long periods of time (Scheffrahn *et al.*, 1987; Knol *et al.*, 2005). Symptoms such as headache, concentration and memory problems, dizziness and nausea, irritation of the skin and mucous membranes, neurological and neuropsychological impairment, and reactive airways dysfunction syndrome have been reported among patients after contact with fumigants (Preisser *et al.*, 2011). Two case reports directly related to work inside containers are described in a recent article by Preisser *et al.* (2012).

Considering the size of the container transportation industry, there are surprisingly few peer-reviewed studies reporting from screening ocean freight containers for toxic substances. In one of these few, just >2000 incoming containers were investigated in the Port of Hamburg during a 10-week period in 2006 (Baur *et al.*, 2010). The most frequent contaminants found were formaldehyde (59%) and benzene (19%) and, among the fumigants, methyl bromide (14%), phosphine (4.5%), and chloropicrin (1.7%).

Among the non-peer-reviewed studies, roughly 300 randomly selected import containers were examined in 2002 in the Port of Rotterdam (Knol-de Vos, 2002). Methyl bromide, formaldehyde, and phosphine were found in 21% of the containers. In 5% of the 300 containers, the levels of these fumigants exceeded the Dutch 8-h occupational exposure limits (OEL). In a study of 50 000 containers in the Benelux container terminals during 2010, volatile chemicals were identified and grouped according to the type of goods transported. The most common chemicals identified were 1,2-dichloroethane, carbon monoxide, formaldehyde, toluene, and benzene at a frequency of ~2% each. Phosphine was present in 0.08% and methyl bromide in 0.06% of the containers (Luyts, 2010).

Personal exposure to 13 selected residual chemicals is reported in a recent hazard surveillance published by the Safe Work Australia (2012). Residual chemicals were detected in 74 of the 76 investigated containers. The most common volatiles were toluene, C2-alkylbenzenes, and methyl bromide. In 8% of the containers, personal peak levels exceeded the Australian national workplace exposure standards (WES) for chloropicrin and formaldehyde. None of 12 personal samples, covering the entire duration of stripping a container (2–3h), exceeded the WES.

We have previously sampled arrival concentrations in 101 randomly selected incoming containers in the Port of Gothenburg, Sweden, in 2010 (Svedberg and Johanson, 2011). Trace amounts of the fumigant carbonyl sulphide were found in one container (1 p.p.m.). Most containers had detectable levels of volatile chemicals; most commonly methanol (78% of the containers), hydrocarbons (47%), carbon monoxide (45%), and ammonia (15%). Of the measurements taken, 7% were above or well above the Swedish 8-h OEL. Aside from representing random variability and true differences, the deviating results in the above studies also reflect different detection limits and other technical limitations in the analytical methods.

In spite of the sometimes high concentrations found in the screening studies, we found no reports in the scientific literature regarding workers’ exposure. The aim of this study was to assess workers’ exposure to volatile chemicals during complete stripping operations and evaluate them in relation to the initial arrival concentrations.

## METHODS

### Study locations

Two major Swedish retail businesses were studied as their import containers were stripped of their content in their respective central distribution warehouse. The containers were positioned as usual with the container doors in the rear facing the inside of the warehouse. Once positioned, a distance of ~0.5–1.0 m free passage to the open air around the perimeter of the container remained. This open space was normally closed during stripping by the use folding curtains operated by pressurized air. In both warehouses, the loading docks were separated from the storage area by large doors that remained closed except during passage.

### Preparation of containers

Only 40-foot containers were included in the study since they are likely to cause a higher exposure than the shorter 20-foot type. The contents of the containers were cartons of various sizes with electronics, tools, shoes, and sporting goods. The containers were filled to an estimated 80–100%. The containers targeted for the study were opened, and three sampling sites were arranged at 12, 6, and 0 m from the door as follows. For the 12-m position, six sections of 2 m × 16mm stiff polyvinyl chloride tubings were fitted in line and positioned in the space between the goods and the container ceiling. Similarly, three sections were fitted for the 6-m position. Immediately inside the doors, the sampling lines were connected to a short 10-mm diameter nylon tube, which snugly fit between the rubber door seals when closed. Only the short nylon tube was used for the 0-m position.

### Tracer gas studies

Although some import containers have sufficient arrival concentrations of off-gassing volatile organic compounds (VOCs), their appearance may be too rare and unpredictable to be practically useful for personal exposure studies. For this reason, we wanted to investigate if an experimental tracer gas method could approximate the behaviour of real contaminants. The tracer gas chosen was nitrous oxide (N_2_O), which was added prior to stripping the container. Nitrous oxide has several advantages: it does not normally occur in containers, it is harmless at the concentrations used, and it has a distinct infrared signal, which can easily be monitored. Nitrous oxide is commonly utilized in ventilation studies as well as in medical applications. The Swedish 8-h OEL for nitrous oxide is 100 p.p.m., and the 15-min short-term OEL is 500 p.p.m. (SWEA, 2011).

After closing the prepared containers, nitrous oxide was injected from a gas cylinder through the 6-m sample line. The application was gauged to reach a final concentration of ~500 p.p.m., however, due to a varying degree of free airspace from one container to another, the target concentration was highly approximate. The tracer gas was let to disperse for at least 24h before the container was ready for stripping. This part of the study was only conducted after approval by the Regional Swedish Ethics Committee and informed consent by the workers involved in container stripping.

### Initial concentrations

Initial concentrations of the tracer gas and any naturally occurring off-gassing chemicals were measured in all three sampling positions immediately before opening the container doors for stripping. Air was pumped directly to a gas cell for measurement with a Fourier transform infrared (FTIR) spectrometer and, using the outlet air from the gas cell, with a direct reading photoionization detector (PID). Alternatively the air was sampled in bags for later analysis by FTIR and PID as well as for collection on adsorbent tubes. Further details on the analytical methods are given below.

### Personal exposure monitoring

Personal monitoring was arranged to determine the average exposure during a complete stripping process. All tested containers were ventilated naturally during stripping through the open container doors. Typically, a 40-foot container was stripped in 2–3h. In one of the companies, two workers unloaded the goods onto pallets, while a third person operated a forklift removing loaded pallets and delivering a stack of empty ones when required. The other company made use of a conveyor belt that extended into the container. Only one person inside the container unloaded goods onto the conveyor.

The persons working inside the container were equipped with backpacks to accommodate equipment for bag and adsorbent tube sampling and for the PID instrument. In case there was a shift of personnel, the equipment was transferred to the new worker. The sampling and monitoring started prior to opening the container doors and continued until the container was completely emptied.

#### Bag sampling (six containers).

A 10-l sample bag (Tedlar®) was placed in the backpack and connected to a sample pump (SKC Model 224-PCXR7) set at a flow rate of 200ml min^−1^, giving an effective sample time of just <50min per bag. The sample tube inlet was positioned in the breathing zone near the workers’ lapel. The bag was replaced when nearly full. Analyses of the contents of the sample bags were done on-site by FTIR in six containers and by PID in two of them.

#### Direct reading PID instrument (three containers).

The PID instrument was placed in the backpack and used in ‘hygiene’ mode for continuous personal monitoring. One-minute average readings were logged. The sample nozzle had a dorsal position slightly above shoulder height.

#### Adsorbent sampling (three containers).

Adsorbent tubes (Anasorb 747) were connected to a sample pump (GSA SG 350ex) set at a flow rate of 333 ml min^−1^. The sample tube inlet was positioned in the breathing zone near the workers’ lapel.

### Work zone monitoring

The work zone was defined as the work area within an arm’s length distance from the worker and is a close approximation of the personal exposure. A diaphragm pump (KNF Type NO26 1.2 AN.18, KNF Neuberger GmbH, Freiburg-Munzingen, Germany) continuously pulled work zone air at an effective flow rate of 5 l min^−1^ through a 10-mm diameter sample line to the FTIR instrument positioned outside the container. The sample line was positioned not to obstruct the workers’ movements and was continually repositioned as the work zone gradually moved towards the front end of the container. Air samples were collected as 30-s to 2-min averages, the shorter times to observe peak exposures during opening of the containers and the longer to enhance the detection limits as the levels tapered off.

### Chemical analyses

#### Fourier transform infrared.

Two FTIR instruments were used in this study; a Bomem MB 100 and a MB 3000 (Bomem Inc., Quebec, Canada). They were equipped with a 1-m, 10-m, or 20-m analytical gas cell, the optical path length depending on application and configuration. Qualitative and quantitative analyses of nitrous oxide and VOCs were based on library spectra from Infrared Analysis Inc. (Irvine, CA, USA). Concentrations >100 p.p.m. of nitrous oxide were quantified by creating a calibration file with a set of spectra based on the Hitran2000 spectral database (Griffith, 1996; Rothman *et al.*, 2003).

#### Photoionization detector.

A hand-held PID (ppbRAE Plus, RAE Systems, San Jose, CA, USA) with a built-in data logger was used to measure the VOCs.

#### Adsorbent tubes.

Analyses were done using gas chromatography-mass spectrometry (GC-MS) technique in scan mode, dichloromethane extraction, and a phenyl-dimethylpolysiloxane column (Eurofins Pegasus lab, Uppsala, Sweden). The VOCs were determined in the 80–300°C boiling point range and expressed as toluene equivalents.

## RESULTS

### Dispersion of tracer gases and legitimacy of the tracer gas method

**Fig. 1.**
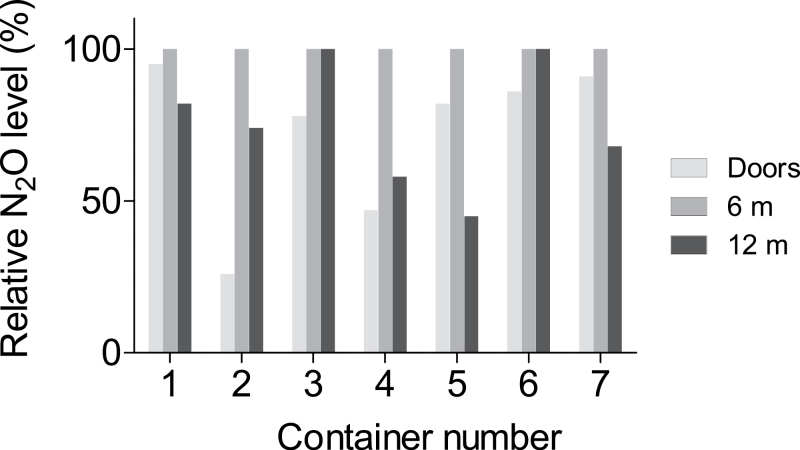
Distribution of nitrous oxide (N_2_O) tracer gas after 22–25h of equilibration in seven containers. The concentrations are expressed relative to that at the site of injection, i.e. at 6 m.

**Fig. 2.**
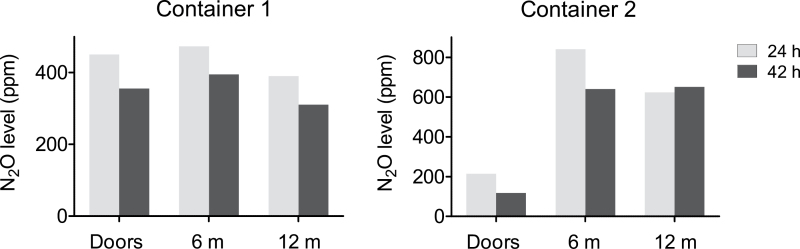
Distribution of nitrous oxide (N_2_O) tracer gas injected at the 6-m position after 24 and 42h of equilibration.

**Fig. 3.**
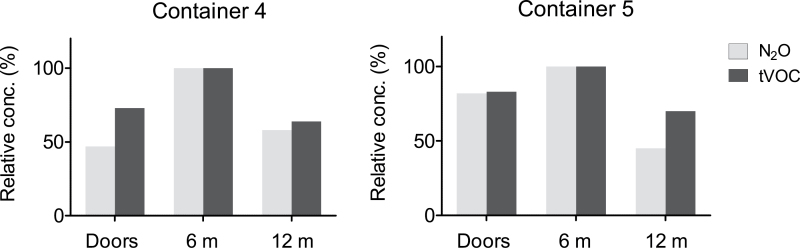
Concentrations of naturally occurring VOCs and nitrous oxide (N_2_O) tracer gas and in two containers after 24h of equilibration of the tracer gas. The concentrations are expressed relative to that measured at 6 m, the site of N_2_O injection.

A well-distributed tracer gas is a precondition for reliable ventilation and exposure studies. The concentration of the tracer gas after 22–25h of equilibration is shown in Fig. 1. As the absolute concentrations are of no interest, they are expressed relative to those measured at 6 m, i.e. at the injection point. In all seven containers, the relative concentration was lower inside the doors (0 m) and in five containers, it was also lower at the front end (12 m). This pattern remained after 42h (tested in two containers, Fig. 2). The distribution of tracer gas resembled that of naturally occurring VOCs as illustrated in Fig. 3 and is an illustration that the tracer gas method well approximates the behaviour seen with off-gassing chemicals.

### Exposure measurements

The measurements of personal and work zone samples in six 40-foot containers are summarized in Table 1. All exposure levels are expressed relative to the initial concentrations recorded inside the doors (0 m) just before opening. This reference point was selected since it is, in the normal handling in container terminals, accessible by inserting a sample nozzle between the rubber seals around the doors. There was some variability between the measurements depending on the sampling and analytical method, but the aggregate results showed personal exposures levels between 1 and 7% of the concentrations in the unopened containers. The personal exposure based on FTIR analysis of nitrous oxide collected in bag samples showed an average exposure of 2.0±0.82% (*n* = 6) of the initial concentrations. The results from FTIR and PID measurements showed good agreement, while the results from adsorbent sampling yielded slightly higher figures of 5.4–6.7%.

**Table 1.** Personal and work zone samples collected during stripping of six containers. The concentrations are expressed relative to the initial concentration just inside the doors before opening the container.

Analyte	Method	Container ID					
		4	5	8	9	10	11
		Stripping time (min)					
		184	90	173	74	110	145
		Relative concentration (%)					
Personal sampling							
VOC	Adsorbent tube	4.7	6.7	5.4	—	—	—
VOC^a^	FTIR bag sample	2.1	1.4	2.8	—	—	—
N_2_O^a^	FTIR bag sample	2.2	1.3	3.4	1.2	1.6	2.3
VOC^a^	PID bag sample	—	1.1	2.1	—	—	—
VOC	PID continuous	1.7	2.7	3.1	—	—	—
Work zone sampling							
VOC	FTIR continuous	0.7	1.2	—	—	—	—
N_2_O	FTIR continuous	1.4	0.7	—	1.3	1.7	1.5
Peak exposure at container opening^b^							
N_2_O work zone	FTIR	3.8	3.7	—	10	2.0	6.3
VOC personal	PID	3.0	70	3.9	—	—	—

—, no data available.

^a^Parallel samples extracted from the same sample bags.

^b^Measured as 0.5-min or 1-min averages.

**Fig. 4.**
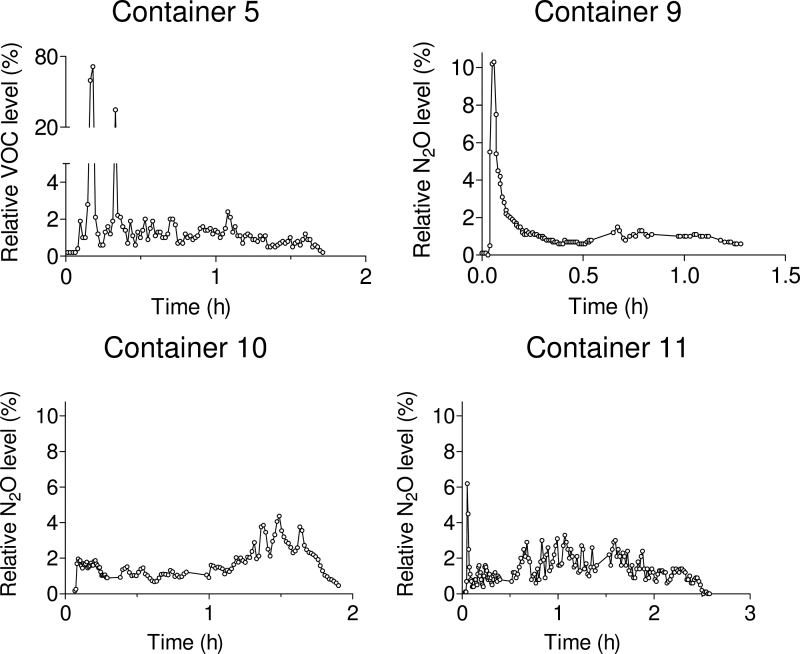
Results from personal monitoring of VOCs by PID (container 5) and work zone monitoring of nitrous oxide (N_2_O) tracer gas by FTIR (containers 9–11). The concentrations are expressed relative to the initial concentration inside the door prior to opening the container. Note the broken *y*-axis of the VOC graph.

The average levels recorded by continuous work zone monitoring showed good agreement with those collected by personal sampling. The graphs in Fig. 4 illustrate representative work zone data monitored by FTIR in three containers (nos 9, 10, 11) and from continuous personal monitoring with the PID instrument in one container (no. 5). Upon opening the containers, peak exposures were occasionally observed. These peaks were usually <10% of the initial concentration (based on 1-min averages) but reached 70% in one case. Disregarding the opening peaks, the readings fluctuated between 2 and 4% throughout the remainder of the stripping operation.

**Fig. 5.**
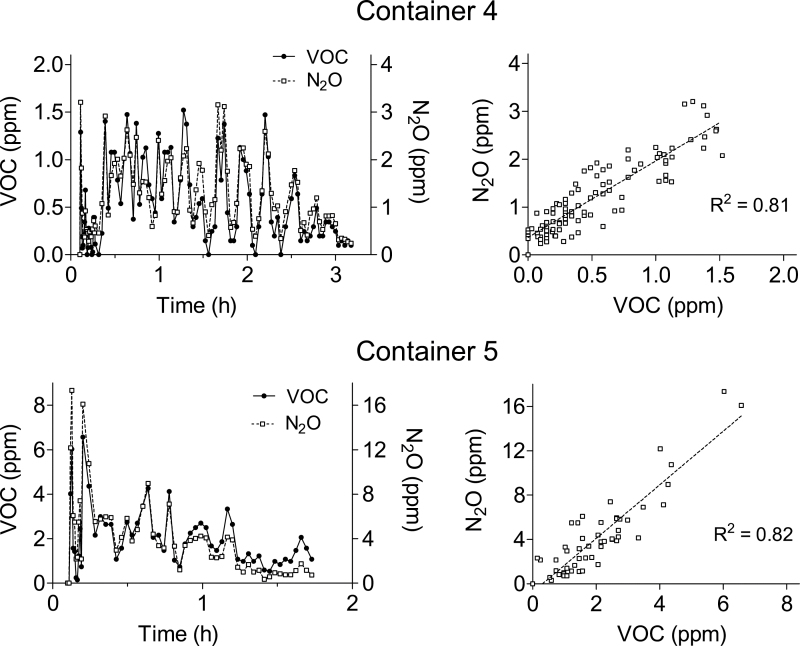
Work zone levels of VOCs and nitrous oxide (N_2_O) tracer gas (left panes) and their correlation (right panes) as measured by FTIR in two containers.

In two containers, both stuffed with boxes with sneakers, the levels of naturally occurring VOCs were sufficient to follow the time-trend during stripping and compare with the behaviour of the tracer gas. When VOCs and the tracer gas were analysed in the same FTIR spectra, they showed comparable time-trends and correlation coefficients (*r* ^2^) of ~0.8, as illustrated in Fig. 5. This is an additional illustration that the use of a tracer gas may well approximate real exposures. In these two containers, the initial concentrations of total VOC_s_ (measured by GC–MS) were 169 and 219 mg m^−3^, expressed as toluene equivalents. The dominating VOC was toluene (91 and 68%, respectively, of the total VOC_s_). Benzene (0.7 and 4 mg m^−3^, respectively), 2-butanone, ethylbenzene, xylene, heptane, cyclohexylmethane and ethyl acetate were also detected.

## DISCUSSION

Based on the findings in this study, we conclude that workers’ average exposure to off-gassing chemicals during stripping of containers is usually significantly lower (1–7%) than might be expected if only the concentration in the unopened container is considered. Still, peak exposures may occur, in our study reaching as high as 70% of the initial concentration. Such episodes raise concerns for acute health effects and justify preventive measures prior to opening. The low average exposures and the brief peak exposures may explain why only a handful of case reports that directly can be related to the work inside freight containers have been reported in the scientific literature. In spite of the few case reports, we suspect that exposure incidents during container stripping are quite common but only occasionally reported to official reporting centres. Under-reporting may explain why sporadic exposure to high and potentially toxic levels of fumigants and off-gassing VOCs has hitherto not been thoroughly investigated by the scientific community. The workers carrying out stripping in our study expressed that the containers frequently carried unpleasant odours that from time-to-time prevented stripping. Such containers were left for natural ventilation before re-entry. Alternatively, workers were instructed to wear respiratory equipment. However, many chemicals lack warning properties and it is difficult to tell from smell only when a factual risk exists.

Our results draw attention to the need to particularly identify high-risk containers and establish routines how they should be handled safely. As an example, in one of the studies listed in the Introduction, 368 p.p.m. phosphine was recorded in one container (Luyts, 2010). An assumed exposure to 18 p.p.m. phosphine during stripping (5% of 368 p.p.m.) would be 60-fold higher than the current Swedish 8-h OEL of 0.3 p.p.m. and is likely to be lethal or life threatening, as suggested by rodent 4-h LC_50_ values of ~10–30 p.p.m. (NRC, 2007). Should a risk container be identified it would thus not be acceptable to allow workers to enter by referring to studies showing that their exposure will be a minor fraction of the concentration in the unventilated container. Furthermore, the frequently observed lower concentrations inside the doors compared with deeper inside the container might lead to an underestimation of the overall exposure during the entire stripping cycle.

A plausible explanation why some containers produce a distinct short peak at the time of opening, while others show none or an extended peak is the filling degree of the container. A fully loaded container is not likely to release large volumes of container air upon opening the doors. Another possibility is that the subject carrying the personal monitoring equipment temporarily stepped away from the container, and the opening peak was not captured by the instruments. The increase in concentrations of tracer gas observed towards the end of the stripping of container 10 in Fig. 4, could possibly reflect the variation of concentrations seen inside containers, as illustrated in Fig. 3.

The saw tooth fluctuations seen in Figs 4 and 5 were likely reflections of the intermittent nature of the work caused by restrictions in the flow chain. Thus, as new groups of boxes were removed, new portions of tracer gas and VOC-containing air were released to the work zone. One might suspect that the fluctuations also reflect the repositioning of the FTIR sample line as the work zone progressively moved into the container. This is contraindicated by the PID measurements, which were carried out at a fixed position in the breathing zone near the mouth and, nevertheless, show a similar variability.

A limitation in this study is that we only measured exposures in containers with boxed cargo. Stripping of other types and formats of cargos, such as sacks, bales, lumber, machine components, or tires, may take longer and could result in higher VOC exposures. Other occupational groups, such as customs inspectors (see Introduction), may encounter much higher exposure levels, even approaching those encountered before opening the container. Additional studies covering other types work tasks and cargos are needed to get a more complete picture.

Altogether, our observations form a strong argument that the tracer gas method to a large extent approximates the real exposure scenario, at least for stripping of boxed goods. The tracer gas method may be the only acceptable experimental approach to estimate personal exposure to fumigants and other highly toxic substances that cannot easily be investigated in experimental exposure studies. Twenty-four hours of equilibration of the tracer gas appears to be sufficient and is also the time recommended by the IMO for the reversed purpose to reach adequate penetration of fumigants into the goods during fumigation (IMO, 2008). The similar concentration profiles between the off-gassing chemicals and the tracer gas (Fig. 3) and similar work zone data (Fig. 5) indicate good penetration of the tracer gas into the goods. The ambient temperature is described by the IMO to affect penetration time during fumigation, and it will likely also affect the equilibration of tracer gases and emission of off-gassing chemicals. The conditions during this field study varied between minimum of −3°C at night and midday temperatures in the 5–15°C range. It is expected that higher ambient temperatures increase the off-gassing resulting in higher initial concentrations. On the other hand, high or variable temperatures may introduce increased air movement in the containers, resulting in more natural ventilation and lower concentrations.

All ocean freight containers have small openings in the top corners to provide limited natural ventilation. These openings and, in addition, possible leaking rubber seals around the doors may explain our observation of an uneven distribution of tracer gas and VOCs. This inhomogeneity may also be caused by air movements in parts of the container, as solar radiation heats one side of the container only. A third explanation may be that high and low emitting goods are stuffed in different parts of the container.

Labelling is mandatory for fumigated containers, i.e. those treated with specific chemical substances defined as fumigants. However, labelling is not required for containers carrying goods that emit other hazardous chemicals, usually as a result of post-production off-gassing. Ideally, containers stuffed with such goods should also be labelled or, better, the volatiles should be eliminated from the products before shipping. Until this has been achieved, the safest practice is to measure every incoming container and pre-ventilate them thoroughly as needed. Monitoring of container air is relatively easily implemented at high volume container terminals but may be more difficult to implement at small-size terminals workplaces and particularly in developing countries. Pre-ventilation is a cost-effective approach that can more easily be made available to any size of the terminal. Although our study suggests that the natural ventilation occurring via the open container doors eliminates a major part of the air pollutants in the work zone, it should be emphasized that pre-ventilation should be carried out in those containers where high concentrations of harmful substances (above the OEL_s_) have been detected.Depending on the type of goods transported, harmful concentrations may be present in a large percentage of the incoming containers. There is thus a pressing need to find technical solutions to facilitate rapid air sampling and efficient ventilation prior to opening the container.

Unfortunately, the current container design makes safe and speedy sampling and ventilation prior to opening the doors technically difficult. This shortcoming tends to promote risky work behaviour (such as using smell as the sole warning signal) to avoid unwanted and costly lag times in the transport logistics. The Australian Customs and Border Protection Service require testing for fumigants by drilling two to three small holes in the container (ACBPS, 2012). The Canadian Border Service Agency (CBSA) has recognized that the procedures used to ensure that a container is safe before entry severely delay the throughput, and they have accordingly initiated a study to alleviate such delays (CBSA, 2008).

We propose that the container manufacturers provide pre-installed ventilation ports at the front end of the container where an external portable extraction fan can be connected and thereby forcing fresh air into the container through the doors left ajar. Such experiments are currently underway and will be reported in due course. We also propose pre-installed internal sample lines that allow sampling of air in the front, middle, and rear of the container. The sample lines would be accessible from the outside at the rear end (door end) of the closed container via through connections. Screening and identification of risk containers could thus be carried out routinely and systematically, even on stacked or tightly stored containers. Resources and preventive actions may then be more effectively directed to the problem containers.

The results from this and previous studies illustrate the need to establish practices for the safe handling of ocean freight containers. Until comprehensive recommendations are in place, those needing to enter such containers should have access to equipment for measuring contaminants and/or applying forced ventilation if necessary. Personal protective equipment should be used, and rescue strategies made available to individuals who need to enter unventilated containers with unknown hazards.

## FUNDING

AFA Insurance (110255); Västernorrland County Council.
